# The other ‘C’: Hospital-acquired *Clostridioides difficile* infection during the coronavirus disease 2019 (COVID-19) pandemic

**DOI:** 10.1017/ice.2021.3

**Published:** 2021-01-13

**Authors:** Karl Hazel, Mairead Skally, Emily Glynn, Margaret Foley, Karen Burns, Aoibhlinn O’Toole, Karen Boland, Fidelma Fitzpatrick

**Affiliations:** 1 Department of Gastroenterology, Beaumont Hospital, Dublin 9, Ireland; 2 ESCMID Study Group for Clostridioides difficile; 3 Department of Microbiology, Beaumont Hospital, Dublin 9, Ireland; 4 Department of Clinical Microbiology, Royal College of Surgeons in Ireland, Dublin 9, Ireland

*To the Editor*—We read with interest the recent article by LeRose et al^1^ on the impact of coronavirus disease 2019 (COVID-19) on healthcare-associated infection. In contrast to their observations of increased central-line–associated infection and blood-culture contamination rates during the first wave of the COVID-19 pandemic, we observed a decrease in hospital-acquired *Clostridioides difficile* infection (HA-CDI) within our institution over this time, compared with the same period in previous years.

CDI is the leading cause of hospital-acquired infectious diarrhea. Risk factors include older age, comorbidities, and most notably, broad-spectrum antibiotic use.^2^ High bed occupancy in acute-care hospitals correlates with an increased incidence of healthcare-associated CDI (HA-CDI).^3^ The COVID-19 pandemic has caused significant changes within the healthcare system worldwide. In hospitals, the cessation of elective procedures in early March combined with an overall reduction in emergency presentations for non–COVID-19–related illnesses led to a reduction in hospital occupancy rates from March to May 2020.^4^ Concern has been expressed that COVID-19 may impact CDI rates, especially in the elderly.^5^ Older people with comorbidities are disproportionately affected by COVID-19.^6^ Concurrent broad-spectrum antimicrobials to treat bacterial co-infection and super-infections in COVID-19 may also increase the risk of CDI.^7^ Conversely, the increased focus on infection prevention and control may prevent cross transmission of *C. difficile*.

We hypothesized that the infection prevention and control measures implemented in our institution to prevent COVID-19 transmission would also influence HA-CDI. These measures included a hospital-wide transmission-based–precautions educational program, increased focus on hand hygiene compliance and audit, social distancing, and reduced ward occupancy.

Our institution is an adult tertiary-care referral center with >800 beds and 136 single rooms (77% with en suite facilities) and 12 airborne isolation rooms. Most accommodation is multi-occupancy; comprising 6-, 4- or 2-bed rooms and shared bathroom. We defined the first COVID-19 wave in our institution as March 1 to May 31, 2020. The first positive inpatient with COVID-19 was admitted on March 10, 2020. Daily on-site SARS-CoV-2 real-time polymerase chain reaction (PCR) testing commenced on March 16 for patients with suspected COVID-19 and for all admitted patients on April 19.^8^ Daily onsite *C. difficile* laboratory testing continued without interruption during the first COVID-19 wave. This involves a 2-step protocol: testing for *C. difficile* toxin B gene tcdB by PCR and if positive, testing for *C. difficile* toxin. Positive results are reported by telephone daily by the clinical microbiologist, who also discusses relevance and recommended management plans. Patients are isolated with contact precautions, and on discharge, hydrogen peroxide decontamination of the area is performed prior to new patient admission.

Data on newly acquired HA-CDI from March 1 to May 31 were collected and compared to the same periods in 2018 and 2019. CDI data were extracted from the hospital CDI database. This database comprises CDI data, which are collected and validated prospectively, with assignment of CDI case type as outlined in national guidelines.^9^ Patient demographics and biochemical markers were collected from the patient administration systems. Hospital antimicrobial consumption and hand hygiene audit data for the same periods were also collected. One-way ANOVA using Prism software (GraphPad, San Diego, CA) was employed to determine whether there was a statistically significant difference between rates of CDI during the pandemic period versus the same periods in 2018 and 2019.

In total, 50 patients with HA-CDI were identified, and most were admitted under the care of medical specialties: 14 in 2018, 27 in 2019, and 9 in 2020 (4 of whom had COVID-19) (Table 1). Compared with the previous 2 years, hospital admissions were lower (*P* < .0001) and hand hygiene audit scores showed a significant improvement during the first COVID-19 wave compared with 2018 (*P* = .0015) and 2019 (*P* = .045), with no change in antimicrobial consumption. We observed a decrease in length-of-stay in 2020, but this was not significant. Newly acquired HA-CDI decreased during the first wave of the COVID-19 pandemic period compared with the same periods in 2018 (*P* = .0013) and 2019 (*P* < .0001) (Table 1).

**Table 1.** Details of Patients With Hospital-Acquired *C. difficile* Infection (HA-CDI), March 1 to May 31, 2018–2020: Hospital Activity, Antimicrobial Consumption and Hand Hygiene Compliance

Variable	2018 (n=14), No. (%)	2019 (n=27), No. (%)	2020 (n=9), No. (%)
CDI rate per 10,000 BDU	2.24	4.24	2.15
COVID-19 infection	0	0	4
Sex, male	5 (35.7)	16 (59.3)	7 (77.8)
Age, mean y (range)	71 (17–93)	68 (31–89)	67 (33–87)
Admitting specialty, medical	10 (71.4)	14 (51.9)	6 (66.7)
Admitting specialty, surgical	2 (14.3)	9 (33.3)	2 (22.2)
Critical care admission^1^	2 (14.3)	4 (14.8)	1 (11.1)
Concurrent/recent antimicrobials^2^	3 (21.4)	22 (81.5)	7 (77.8)
**Hospital data**			
Hospital admissions	6,368	6,519	4,781
Average length-of-stay, d	9.79	9.73	8.41
Hand hygiene compliance	85	86	90.3
Hospital antimicrobial consumption, DDD/100 BDU	94.5	93	95

Note. BDU, bed days used; DDD, defined daily dose.

1 Patient an inpatient in the critical care unit at time of diagnosis of CDI.

2 Antimicrobial therapy during current admission.

During the first wave of the COVID-19 pandemic in our institution, despite concerns regarding its impact on antimicrobial stewardship, antimicrobial consumption remained stable, with a reduction on HA-CDI compared to the previous 2 years. It is likely that reduced occupancy, length-of-stay, and increased emphasis on infection prevention and control, especially hand hygiene, also played a role. The interplay between the gut microbiome, COVID-19, and *C. difficile* has yet to be elucidated and the impact of COVID-19 on colonization resistance and risk of future CDI unknown. During additional waves of the pandemic, it is essential that CDI prevention, control and management play larger parts in the healthcare response, especially in elderly patients. Unlike the first wave, hospital activity has returned to normal levels, with full bed occupancy. Therefore, vigilance for cross infection, including HA-CDI, is of paramount importance.
